# Characterization of a Microbial Consortium for the Bioremoval of Polycyclic Aromatic Hydrocarbons (PAHs) in Water

**DOI:** 10.3390/ijerph15050975

**Published:** 2018-05-13

**Authors:** Esmeralda G. Blanco-Enríquez, Francisco Javier Zavala-Díaz de la Serna, María del Rosario Peralta-Pérez, Lourdes Ballinas-Casarrubias, Iván Salmerón, Héctor Rubio-Arias, Beatriz A. Rocha-Gutiérrez

**Affiliations:** 1 Facultad de Ciencias Químicas, Universidad Autónoma de Chihuahua, Campus Universitario #2, Circuito Universitario, Chihuahua, Chihuahua C.P. 31125, México; e.blanco2015@hotmail.com (E.G.B.-E.); mperalta@uach.mx (M.d.R.P.-P.); lourdes.ballinas@gmail.com (L.B.-C.); isalmeron@uach.mx (I.S.); 2 Facultad de Zootecnia y Ecología, Universidad Autónoma de Chihuahua, Periférico. R. Almada, Km.1. Chihuahua, Chihuahua C.P. 31453, México; rubioa1105@hotmail.com

**Keywords:** consortium, PAHs removal, SPME-GC/MS, water pollution

## Abstract

Pollution of freshwater ecosystems from polycyclic aromatic hydrocarbons (PAHs) is a global concern. The US Environmental Protection Agency (EPA) has included the PAHs pyrene, phenanthrene, and naphthalene among the 16 priority compounds of special concern for their toxicological effects. The aim of this study was to adapt and characterize a microbial consortium from ore waste with the potential to remove these three PAHs from water. This microbial consortium was exposed to the target PAHs at levels of 5, 10, 20, 50, and 100 mg L^−1^ for 14 days. PAH bioremoval was measured using the analytical technique of solid phase microextraction, followed by gas chromatography mass spectrometry (SPME-GC/MS). The results revealed that up to 90% of the target PAHs can be removed from water after 14 days at a concentration level of 100 mg L^−1^. The predominant group of microorganisms identified at the phylum taxonomic level were the Proteobacteria, while the Actinobacteria were the predominant subgroup. The removal of phenanthrene, naphthalene, and pyrene predominantly occurred in specimens of genera *Stenotrophomonas*, *Williamsia*, and Chitinophagaceae, respectively. This study demonstrates that the use of specific microorganisms is an alternative method of reducing PAH levels in water.

## 1. Introduction

During the last few years, an increase in organic contaminants in water due to accelerated development and industrialization growth has been detected. Many of these contaminants are considered potentially toxic to the environment and to human health. For that reason, water sheds contaminated with organic contaminants have gained considerable worldwide concern [1,2].

One type of these organic contaminants is polycyclic aromatic hydrocarbons (PAHs), which have caught scientists’ attention due to their mutability, carcinogenicity, and toxicity. Such compounds originate from oil spills and from incomplete combustion. They are prone to biomagnification in addition to long-distance transport [3].

Other possible PAH sources are agriculture, agricultural runoff, and wastewater from industrial plants. These sources play an important role in water supply and irrigation; therefore, water quality is strongly related to health. Nevertheless, available information on public risk in relation to water quality is very limited [4]. Despite these compounds’ low water solubility, they can be found in concentrations at the µg L^−1^ (ppb) level. Such levels are considered toxic, hence the relevance of their quantification [5].

In addition, PAHs are difficult to eliminate from natural matrices [6]. Physicochemical treatments, such as steam distillation, solidification, chemical precipitation, and incineration, have been implemented in efforts to eliminate these contaminants. However, these methods tend to transport contaminant compounds from one place to another rather than eliminate them. In addition, these treatments can involve other economic costs due to the generation of toxic byproducts that require additional treatment [7]. On the other hand, bioremediation initiatives offer enduring solutions. Such methods are deferential to the environment and have low operation costs, and are therefore supposed as an alternative means of eliminating such contaminants from water [8,9]. Because of the discovery that certain microorganisms are able to grow by utilizing PAHs as a source of energy and carbon, bioremediation is now considered a valuable tool. Bioremediation also leads to the complete organic contaminant mineralization into carbon dioxide and water, or a transformation of complex organic contaminants into other, simpler compounds through certain biological agents, such as microorganisms [10,11].

Another advantage of employing microbial consortia is their large-scale production, since pure cultures are not often economically feasible due to high production costs, substrates, and biomass recovery. Additionally, many microorganisms are not isolated in natural environments but are, instead, part of consortia [12].

However, working with microbial consortia requires an adaptation period, which consists of pre-enrichment and enrichment phases that depend on the compound analyzed, and the microorganisms’ ability to degrade the target PAHs at contaminated sites must be proven [13,14,15,16]. In the present study, ore waste was used because it commonly contains microorganisms that have survived mining contaminants (including PAHs) and that may have the capacity to remove certain organic compounds.

If the microorganisms in a microbial consortium are to be used to degrade specific contaminants, their composition must be identified [17]. Two widely used techniques to identify the composition of microbial consortium members are PCR amplified gene markers, such as 16S rDNA, and metagenomics. Through these techniques, genome sequences of different microorganisms in a community are obtained by extracting and analyzing their DNA on a global scale [18]. Next-generation sequencing (NGS), such as Illumina, allows for massive sequencing. Therefore, the amplification of small yet highly 16S rDNA variable regions has resulted in the deep sequencing of microbial communities. This has enabled the identification of low abundance populations; hence, these NGS methods have been implemented to successfully characterize bacterial communities in sea water, soil, human microbiomes, and wastewater [19].

Accordingly, this study evaluated and characterized a microbial consortium obtained from ore waste at the San Antonio mine, Chihuahua, Mexico, for the purposes of PAH removal from water. This was done through the first metagenomics analysis based on sequencing results from the 16S rRNA region by utilizing Illumina MiSeq. The aim of this work was to adapt and characterize a microbial consortium from ore waste with the potential to remove the PAHs pyrene, phenanthrene, and naphthalene from water.

## 2. Materials and Methods

### 2.1. Site Description and Materials

The ore waste sample was collected from the San Antonio mine located in Santa Eulalia Chihuahua, Mexico (latitude: 28°36′17″ N; longitude: 105°48′40″ W). This mine was one of the main national suppliers of lead, silver, and zinc until 2010. The samples were collected according to Mexican environmental procedure NOM-021-RECNAT-2000 [20]. The samples were acquired aseptically from a layer 15 cm deep and placed in sterilized sealable polythene bags. The samples were later filtered through a 0.850 mm pore size sieve and stored at 25 °C for microbial analysis and selection for the microbial consortium with the potential to remove PAHs.

Naphthalene was acquired from Sigma-Aldrich (St. Louis, MO, USA). Phenanthrene and pyrene were supplied by Columbia Organic Chemicals Co. (Cassatt, SC, USA), and the chrysene-d12 internal standard was purchased from AccuStandard (New Haven, CT, USA). The acetone employed was chromatography grade (J.T. Baker, Avantor Performance Materials, LLC, Center Valley, PA, USA). Ultrapure water was obtained from a FESTA-ULTRAPURE water production system (model UP 09 99 2017, Chihuahua, Mexico). The manual SPME holder and 100 PDMS fibers were purchased in Supelco (St. Louis, MO, USA). The SPME fiber was conditioned as recommended by the manufacturer prior to use. The growth of selected strains for PAHs was prepared using the Lebac medium with the following composition: 2.24 g L^−1^ MgSO_4_·7H_2_O; 2.3 g L^−1^ K_2_HPO_4_; 5.7 g L^−1^ KH_2_PO_4_; 7 g L^−1^ (NH_4_)_2_SO_4_, and 1.0 g L^−1^ yeast extract. Analytic reagents for bioremoval were used.

### 2.2. Enrichment and Selection for the Microbial Consortium Removal of Phenanthrene, Pyrene, and Naphthalene

The adaptation process of the microorganisms using PAHs as a source of carbon and energy was carried out in a 250 mL Erlenmeyer flask containing 50 mL of a sterile Lebac medium. We then added 1 mL of diesel and 1 g of ore waste sample. The diesel was used due to the target analytes present in the hydrocarbons contained in this medium [21]. The adaptation experiment began with a month-long sub-culture. After two sub-cultures, yeast extract was removed from the Lebac medium to force the microorganisms to use the diesel as a source of carbon; sub-cultures were then taken every 15 days for two months. All cultures were incubated at room temperature. After four sub-cultures, the diesel was replaced by the target PAHs (pyrene, naphthalene, and phenanthrene) in the Lebac medium, which was the only carbon source available for the growth and maintenance of the microorganisms, at a concentration of 5 mg L^−1^ [14,22]. The microbial consortium was reserved for the bioremoval experiment.

### 2.3. Bioremoval Experiment

Each microbial consortium obtained from the enrichment experiment was gradually exposed to concentrations of 5 mg L^−1^, 10 mg L^−1^, 20 mg L^−1^, 50 mg L^−1^, and 100 mg L^−1^ of each target PAH (phenanthrene, naphthalene, and pyrene). Each sample was prepared in triplicate. The culture medium was prepared following the methodology reported by Janbandhu and Fulekar [22]. One milliliter of a sub-cultured microbial consortium was used to inoculate 100 mL of Lebac medium (with no yeast extract added) supplemented with a target PAH in a 250 mL Erlenmeyer flask. The inoculated flasks were kept in an orbital shaker incubator at 120 rpm at room temperature (22 ± 2 °C) for 14 days. Abiotic controls were prepared under the same conditions but contained only one of the target PAHs at the appropriate concentration and the Lebac medium. After 14 days of incubation, 50 mL of each culture medium were taken and centrifuged (5804R centrifuge, Eppendorf, Hamburg, Germany) at 10,000 rpm for 10 min. The supernatant was used for the SPME sample preparation, and the biomass was reserved for DNA extraction.

### 2.4. Sample Preparation and PAH Extraction

The most common methods for PAH extraction in water include liquid–liquid extraction (LLE) and solid phase extraction (SPE). In either extraction method, additional steps to purify and concentrate the analytes are tedious, time-consuming, and in some cases not environmentally friendly due to the large amounts of solvent employed throughout the process.

The solid phase microextraction (SPME) is a solvent-free technique that offers sensitivity, quickness, and robustness for organic compounds, such as the PAHs in this study. A small volume of sample is used to isolate the analytes from the matrix and concentrate them in a fiber phase made of materials (stationary phases) developed in accordance with polarity principles. The fiber remains in contact with the sample until equilibrium is reached in the system. After the analytes are adsorbed in the fiber in the extraction step, the fiber is desorbed in an analytical instrument such as a chromatograph for separation and quantitation of the target analytes [23,24,25,26,27].

SPME coupled with gas chromatography mass spectrometry (GC/MS) has been reported as a powerful technique for monitoring organic compounds in food, in environmental matrices, and in forensic and biological applications [23,28]. A 100 μm polydimethylsiloxane (PDMS) fiber has been used to analyze persistent organic pollutants, such as the PAHs at trace levels achieving up to 100% recovery in aqueous samples [25]. Based on this evidence, the SPME-GC/MS method was selected for the analysis of the target PAHs in this study.

The water samples were prepared by pouring 4500 µL of ultrapure water with 400 µL of the supernatant obtained from the bioremoval experiment, which contained one of the target PAHs and 100 µL of internal standard (chrysene-d12) at a final concentration of 30 µg L^−1^ in a 15 mL headspace vial. Vials were sealed with aluminum caps furnished with polytetrafluoroethylene-faced silicone septum and immersed in a sand bath to reach and maintain a temperature of 60 °C. After 5 min of stabilization, the SPME fiber was exposed to agitation at 1000 rpm in the headspace mode for 45 min. After the extraction step, the fiber was immediately desorbed into the injector of the gas chromatograph (GC) at 260 °C for PAH analysis.

### 2.5. Instrumental Analysis

The PAHs were analyzed in a gas chromatograph (mod. 7890b, Agilent Techologies, Santa Clara, CA, USA) coupled with a mass spectrometer (Agilent Technologies 5975C). The instrument was programmed in the electron impact mode (EI 70 eV) with a cycle time of 2.5 s. The separation was performed using a 30 m × 0.25 mm I.D TG-5HT column (Thermo Fisher Scientific, Swedesboro, NJ, USA) coated with 5% diphenyl/95% dimethyl polysiloxane (film thickness of 0.25 µm). The analysis was programmed using splitless mode, and high-purity (99.99%) helium was employed as the carrying gas at a rate of 1 mL min^−1^. The column temperature was programmed for 80 °C for 1 min at a rate of 15 °C min^−1^ until 280 °C was reached, and this temperature was held for 3 min. The mass spectrometer (MS) source and MS quad temperatures were set at 230 and 150 °C, respectively. The injection volume to identify each compound was 1 µL in SCAN mode. Once PAHs and internal standard were identified, samples were quantified by selective ion monitoring (SIM), scanning the main ions of *m*/*z* 128 (naphthalene), 178 (phenanthrene), 202 (pyrene), and 240 (chrysene-d12). The program runtime was 17.33 min. All analyses were performed in triplicate.

### 2.6. Microbial Consortium Identification

The metagenomic DNA were extracted from culture ore waste and from the three consortia obtained at the end of the cultures (100 mg L^−1^) with phenanthrene, pyrene, and naphthalene, severally. For the extraction, we worked with the methodology of Hoffman and Winston with modifications [29]. A volume of 1400 μL of each culture sample was placed in Eppendorf tubes and then centrifuged (Eppendorf centrifuge 5804 R) at 10,000 rpm, and the supernatant was then discarded. This procedure was repeated until an adequate amount of biomass was obtained (±0.5 mL) for DNA extraction. Then, 150 μL of extraction buffer (Triton 100 × 2%, SDS 1%, 100 mM NaCl, 10 mM Tris base, 1 mM EDTA) were added to the biomass, and the mixture was shaken in a vortex mixer (Maxi Mix plus, Thermolyne, Thermo Fisher Scientific, Waltham, MA, USA) for 1 min. After this procedure, 100 μL of sterile water was added to the tubes and shaken again for 30 s. The samples were centrifuged at 10,000 rpm for 10 min at 4 °C. The supernatant was transferred to a new tube and phenol chloroform isoamyl alcohol (25:24:1) was added at a 1:1 (*v*/*v*) ratio. This mixture was stirred manually for 5 min and then centrifuged at 10,000 rpm for 10 min. The aqueous phase was transferred to a new Eppendorf tube and chloroform isoamyl alcohol (24:1) was added at a 1:1 ratio (*v*/*v*) with the recovered sample. The sample was then stirred manually for 1 min, and all samples were centrifuged under the same conditions described before. The aqueous phase was transferred to a new tube and isopropanol was added in a 1:1 ratio (*v*/*v*). This mixture was shaken gently and incubated at −20 °C for 24 h. Finally, the samples were centrifuged at 10,000 rpm for 12 min at 4 °C, the supernatant was then discarded, and 500 μL of 70% ethanol was then added. The samples were centrifuged at 10,000 rpm for 6 min and the supernatant was removed. DNA pellets were dried at room temperature before they were dissolved in 25 μL of sterile water. The samples were stored at −20 °C. The DNA of the samples was sent Macrogen (Seoul, South Korea; www.macrogen.com), to be sequenced. The target amplified was V4-V5 region of the 16S rRNA genes. The sequencing was performed using the Illumina MiSeq System [30].

### 2.7. Data Analysis

The sequence reads generated from Illumina MiSeq System were processed using the Quantitative Insights Into Microbial Ecology (QIIME) software (version 1.9.1). All reads were reviewed and trimmed for sequences with more than 36 bp (base pairs), and reads with less than 36 bp were discarded. After filtering the reads, they were grouped into Operational Taxonomic Units (OTUs) using an open reference OTU selection protocol. The OTUs were compared to the UCLUST database, based on a sequence similarity of 97%, and then grouped by different taxonomic levels with the workflow script summary_taxa_through_plots.py.

The chimeras were eliminated using the UCHIME removal method implemented in the tool USEARCH, and reads were revised with the USEARCH tool [31,32]. After filtering the reads, the average size was of 256 bp, and these were used to identify the microbial consortia.

For all reads analysis, the Python scripts in the QIIME software was used to determine the “alpha_rarefaction.py” workflow script. This script calculated the alpha diversity, or the within-sample diversity, and generated rarefaction curves (graphs of diversity vs. sequencing depth) using OUT tables. In addition, this script was used to compute different alpha diversity metrics (Observed species, Chao1, Simpson and Shannon) for different samples.

Beta diversity between different samples was computed using the workflow script “beta_diversity_through_plots.py”. This diversity represents a comparison of microbial communities based on their composition. The script was used to generate beta diversity analysis, the Unweighted Pair Group Method with Arithmetic Mean (UPGMA) tree, and principal coordinates analysis (PCoA). The chart were constructed using unweighted UniFrac distance matrices for beta diversity analysis [33]. The generated 3-dimensional (3D) PCoA plots were then visualized using the “Emperor” tool [34].

## 3. Results and Discussion

### 3.1. Removal of PAHs through the Use of Microorganisms from Ore Waste

**Figure 1:**
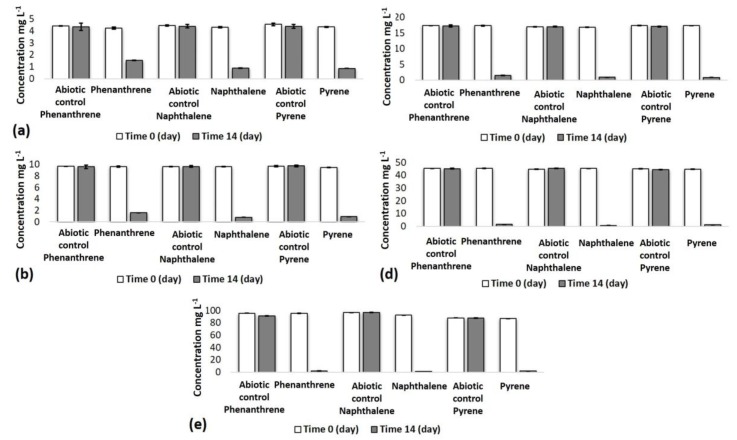
Bioremoval of polycyclic aromatic hydrocarbons by microbial consortium. (**a**) 5 mg L^−1^; (**b**) 10 mg L^−1^; (**c**) 20 mg L^−1^; (**d**) 50 mg L^−1^; (**e**) 100 mg L^−1^. The relative standard deviation (RSD) was less than 20% in all samples. Samples were analyzed in triplicate.

In the present study, three microbial consortia were obtained from ore waste. These consortia showed a removal up to 90% after 14 days of culture in concentrations of 5, 10, 20, 50, and 100 mg L^−1^ of, individually, phenanthrene, pyrene, and naphthalene. The results were compared to the abiotic controls lacking microorganisms to demonstrate the effectiveness of the consortia’s bioremoval (Figure 1).

Previous studies obtained similar removal rates of PAHs but at lower concentrations. Sánches et al. obtained a removal of 100% phenanthrene after 15 h of incubation to 0.1 mg L^−1^ [35]. Other researchers such as Bautista et al. have worked with consortia obtained from samples that were continuously exposed to PAHs, and they reported 100% removal of phenanthrene, naphthalene, and anthracene after a period of 30 days using a consortium from a soil sample that had been chronically exposed to petrochemical products [36]. Huijie et al. reported a 90% removal for phenanthrene and pyrene at a concentration of 50 mg L^−1^ within 20 days of incubation. This removal was obtained with an isolated consortium from a mangrove, whose site was exposed to PAH contamination [37]. Even though the ore waste sample used in this study was not obtained from sites constantly exposed to PAHs, as opposed to the other studies, a removal of at least 90% was still reached. In a similar study performed by Ferradji et al. a consortium was adapted to a hydrocarbon, and an 80% naphthalene removal at a concentration of 100 mg L^−1^ within 12 days of incubation was reported [38]. This result was lower than the 99% naphthalene removal reported at the same concentration after 14 days of incubation in the present study. This shows that bioremediation through microbial consortia isolated from ore waste may be an alternative for the elimination or reduction of low-molecular-weight PAHs in water.

### 3.2. Sequencing Results and Diversity Index

**Figure 2:**
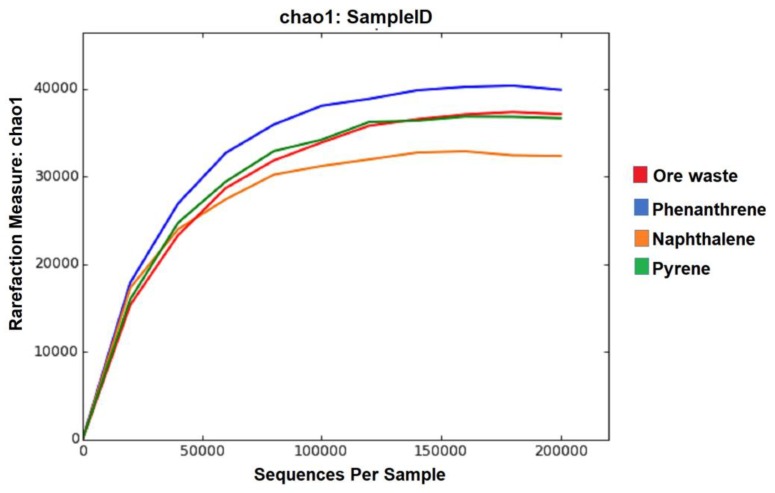
Rarefaction curves showing observed species richness of the four samples: ore waste (OW), phenanthrene (PHE), naphthalene (NAP), and pyrene (PYR).

A total of 1,522,910 high-quality reads with a range of 365,000–390,000 per sample and 633 OTUs were obtained after eliminating incomplete and flawless sequences. The rarefaction analysis (Figure 2) plots the saturation of 250,000 reads and demonstrates appropriate sampling.

The microbial consortium showed a high removal capacity (over 90% for pyrene, naphthalene, and phenanthrene) at a concentration of 100 mg L^−1^ in water and after 14 days of incubation. The studied alpha indexes, Chao1, Shannon, and Simpson, show a significant diversity supported by richness and relative abundance. As presented in Table 1, Shannon values range from 6.55 to 7.71, and Simpson values range around 0.92–0.95. Among the four samples, a closer relationship is observed between the original sample and the consortium that was stabilized with phenanthrene. Both ore waste and consortium stabilized with phenanthrene and obtained the highest indexes compared to those of naphthalene and pyrene (Table 1).

In addition, the consortium stabilized for naphthalene showed a majority group of Gram-positive bacteria such as Actinobacteria; for the consortium stabilized for pyrene, phenanthrene, and the ore waste, the’ most abundant group consisted of Gram-negative organisms.

This finding may be attributed to the naphthalene’s toxicity. This compound is more soluble than phenanthrene and pyrene, which makes it a more available carbon and energy source for certain types of microorganisms. Herrera et al. mentions that the toxicity of PAHs is not only related to an increase in concentration but also to the chemical structure. The lower-molecular-weight PAHs have a higher solubility in water than do the heavier PAHs [39]. Since the molecular weight of naphthalene is lower than the other two PAHs studied, the lowest richness of the microbial consortium was obtained in this sample, indicating that more specialized consortia is required to degrade this compound.

Other research studies have reported lower species richness in different soil sample sites contaminated by mining activity. Hong et al. obtained lower diversity in a microbial consortium from mining soil samples, reporting a Chao index of 1091–1522 [40]. This index is lower to the reported in this study (32,348–39,895). Kim et al. obtained a richness of 334 OTUs in a consortium from marine coastal invertebrates of South Korea [41]. This value is lower than that achieved in this study (633 OUTs). The alpha indexes in Table 1 demonstrate the high genus diversity that can be found in the ore waste sample, which can be reflected in a higher metabolic diversity than the one expected.

**Figure 3:**
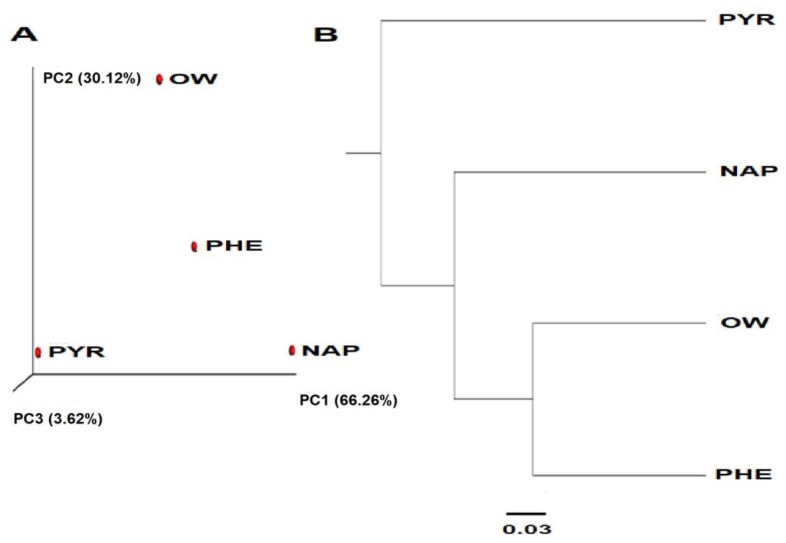
Beta diversity analysis of unweighted UniFrac distance scores. (**A**) PCoA plot and (**B**) UPGMA tree: ore waste (OW), phenanthrene (PHE), naphthalene (NAP), and pyrene (PYR).3.3. Taxonomic Category of the Identification of the Microbial Consortium Exposed to the Different PAHs through the QIIME Analysis

Beta indexes with total variance values for microbial communities of 3.62% for pyrene, 30.12% for ore waste, and 66.26% for naphthalene, through UPGMA and PCoA analysis and through weighted UniFrac analysis, confirmed the distinct community structure of the consortium for each PAH with respect to the ore waste, and showed consistency among the analysis (Figure 3). The most diverse sample came from the ore waste itself. The next sample most related to the ore waste was the sample with phenanthrene, whereas naphthalene and pyrene have a distinct community structure and are not as related to each other. This confirms that each carbon source, with respect to the target PAHs, performs a selective pressure on the microbial community, as was pointed out by Hong et al. [40], Sawulski et al. [42], and Wei et al. [43].

The taxonomic classification of the existing organisms in the consortium was obtained by means of UCLUST (Ultra-fast clustering) [33] using QIIME. All sequences belong to Archaea and Bacteria domains. In Archaea, one phylum was represented; in Bacteria, sequences were affiliated to 16 phyla (Table 2).

Table 3 contains the dominant phyla in each sample of PAH compared to the ore waste sample. As has been mentioned in relation to beta indexes, the consortium most similar to the ore waste sample was the one cultivated with phenanthrene. However, the microbial consortium of phenanthrene presented different relative abundance percentages for the phyla. Pyrene induced a significant difference in the relative abundance of the phylum Bacteroidetes; while there is a small presence in the phenanthrene and ore waste samples (0.01 and 0.05% of the total, the third most abundant phylum in both cases), there is a greater abundance in pyrene (44.6% of the total).

Regarding naphthalene, the difference is even more significant. The dominant phylum in the other two consortia exposed to phenanthrene and pyrene were the Proteobacteria (92.6% for the ore waste, 72.7% for phenanthrene, and 53.5% for pyrene), whereas in naphthalene the dominant phylum was the Actinobacteria (95%). This is relevant since the Proctobacteria phylum is Gram-negative bacteria, while the Actinobacteria are Gram-positive. The fact that naphthalene holds Gram-positive organisms as dominant may be supported by Echeverri et al. who identify these bacteria as important PAHs degraders because the presence of lipoteichoic acid is equivalent to lipopolysaccharides in the Gram-negative bacteria in their membranes, which facilitates the formation and stabilization of hydrocarbon emulsions in aqueous systems and contributes to the increase in the attacking surface of the pollutant [44].

In similar studies on phenanthrene and pyrene removal, such as that by Huijie et al. the dominant phylum was also Proteobacteria [37], which has been reported in wastewater treatment plants (WWTPs), while Bactroidetes and Acidobacteria have been reported as subdominant [45].

**Figure 4:**
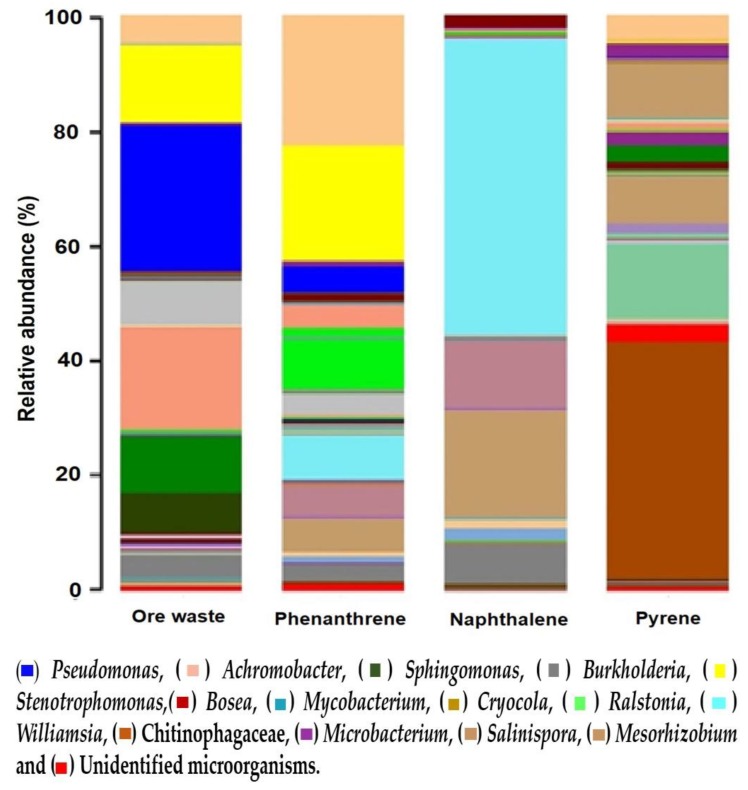
Taxonomic category at the genera level through the bioinformatics QIIME analysis of the sequences obtained by Macrogen.

The taxonomic classification of the four samples (ore waste, phenanthrene, naphthalene, and pyrene) at the genus level is shown in Figure 4. It has been mentioned that the removal of complex PAHs requires the cooperation of more than one species, since an individual microorganism can only metabolize a limited spectrum up to a certain point. Every species performs a different metabolic role, bringing together a general enzymatic ability, otherwise unreachable for a single species [44].

The dominant group in the ore waste sample at the genus level was *Pseudomonas,* Gram-negative bacteria that belong to the phylum Proteobacteria, at 25.28%. *Pseudomonas* are producers of biosurfactants such as rhamnolipids, which are involved in the removal of oils and related products, and extracellular biosurfactants. The production of biosufactants solubilize and facilitate the penetration of hydrocarbons through the hydrophilic cell wall. Furthermore, this genus also contains hydrocarbon-degrading enzymes on the cytoplasmic membrane [46]. *Mycobacterium* has also been found in a PAH-contaminated soil from a natural gas plant, and pyrene was the only source of carbon and energy [47]. These bacteria were also present in the pyrene sample of this study. Echeverrri et al. hold that microbial communities in contaminated areas, such as hydrocarbon-contaminated environments, are dominated by those organisms capable of taking advantage of or surviving toxic compounds. For instance, *Pseudonoma* has been isolated in oceanic and coastal environments. Furthermore, marine bacteria capable of degrading hydrocarbons, called professional hydrocarbonoclastic bacteria (Gammaproteobacteria), have also been isolated [13].

The percentages for the main genera are shown in Table 4. The genera *Sphingomonas* and *Mycobacterium* have been reported as PAH degraders specifically for three- and four-ring structures [43]. *Sphingomonas* utilize PAHs as a sole source of carbon and energy. *Sphingomonas* have catechol 2,3-dioxygenase activity for phenanthrene and anthracene [46]. These bacteria were presented in all four samples in this study.

*Williamsia* has been reported to have potential applications, including bioremediation and biodegradation. Soler et al. isolated this bacteria from wastewater, and the bacteria was observed to use naphthalene as the only carbon source [48]. In the present study, *Williamsia* was the dominant genus in the sample exposed to naphthalene, with a 51.2% presence.

Chitinophagaceae was another genus that appeared as a dominant group. Chitinophagaceae are Gram-negative bacteria that have been isolated from freshwater sediments [49]. This genus was found to possess the capacity to remove PAHs pyrene and benzo[a]pyrene. Chitinophagaceae was isolated from a soil sample from a gas station contaminated with oil [50]. In this study, Chitinophagaceae was dominant with 40.9% for pyrene. Moreover, several authors [36,51,52] have conducted research presenting *Stenotrophomonas* as excellent candidates for PAH removal, particularly phenanthrene. Table 4 shows *Stenotrophomonas* as the dominant group in the phenanthrene sample with a 23% presence. This genus has been isolated in soil from a site contaminated with oil, as well as from marine ecosystems, and are biosurfactant producers. Due to this property, these are compounds with surface activity that contributes to making PAHs available to microorganisms in an aqueous medium, enhancing the removal of hydrophobic substrates. Additionally, dioxygenation in multiple positions is common in the metabolism of *Stenotrophomonas*. Dioxigenation occurs in phenanthrene’s carbon positions: 1, 2, 3, 4, and 9, 10. Comparing the results of the present study to those available in the literature, it is evident that a wide catabolic capacity to remove PAHs is common among all strains due to the structural similarity of many PAHs and the broad enzyme dioxygenase substrate’s specificity.

## 4. Conclusions

The microbial consortium used in this study showed a high removal capacity (over 90% for pyrene, naphthalene, and phenanthrene) at a concentration of 100 mg L^−1^ in water and after 14 days of incubation. To the authors’ knowledge, this is the first study that proposes isolating a microbial consortium from ore waste for the removal of PAHs from water.

The dominant group of microorganisms identified from the microbial consortium at the phylum taxonomic level was Proteobacteria, and Actinobacteria was the dominant subgroup. It must be noted that, depending on the pollutant, the abundance of organisms changes. *Pseudomonas* were found to be the dominant group in the ore waste sample. The dominant group exposed to phenanthrene was *Stenotrophomonas*, that exposed to naphthalene was *Williamsia*, and that exposed to pyrene was Chitinophagaceae. These consortia can be valuable sources of bacteria for the purpose of remediation of other sites contaminated with these PAHs.

## Figures and Tables

**Table 1:** MiSeq sequencing results and diversity estimates for each sample: ore waste (OW), phenanthrene (PHE), naphthalene (NAP), and pyrene (PYR).

Sample		Diversity Indexes		
	Reads	Chao	Shannon	Simpson
OW	390,118	37,123	7.37	0.9576
PHE	365,294	39,895	7.71	0.9508
NAP	390,788	32,348	6.55	0.9426
PYR	376,710	36,657	6.83	0.9249

Chao: Chao’s species richness estimator, Shannon: Shannon–Weiner Index.

**Table 2:** Microbial characteristics of ore waste.

Microorganisms	Phylum
Archaea	Crenarchaeota
Bacteria	Thermi
	Acidobacteria
	Actinobacteria
	Armatimonadetes
	Bacteroidetes
	Chloroflexi
	Cyanobacteria
	Fibrobacteres
	Firmicutes
	Fusobacteria
	Gemmatimonadetes
	Planctomycetes
	Proteobacteria
	Synergistetes
	Tenericutes
	Verrucomicrobia

**Table 3:** Percentage of the dominant phyla in the different samples.

Phylum/Sample	Ore Waste	Phenanthrene	Naphthalene	Pyrene
Proteobacteria	92.6	72.7	4.3	53.5
Actinobacteria	5.5	25.8	95	0.4
Bacteroidetes	0.5	0.01	0.2	44.6
Unidentified	1.1	1.4	0.4	1.2

**Table 4:** Percentage of the genera dominant in the samples.

Genus/Sample	Ore Waste	Phenantrene	Naphthalene	Pyrene
*Pseudomonas*	25.3 *	4.7	0.2	0.01
*Achromobacter*	18.1 *	0.5	0.01	1.1
*Sphingomonas*	9.8 *	0.4	0.01	2.7
*Burkholderia*	7.4 *	3.8	0.1	0.1
*Stenotrophomonas*	5.5 *	23.0 *	0.1	4.6 *
*Ralstonia*	0.1	9.0 *	0.4 *	0.0
*Williamsia*	0.01	8.1 *	51.2 *	0.1
*Cryocola*	0.2	5.9 *	18.9 *	0.01
*Microbacterium*	0.1	5.7 *	11.8 *	0.01
*Serratia*	0.2	1.0	2.6 *	0.01
Chitinophagaceae	0.01	0.01	0.01	40.9 *
*Salinispora*	0.01	0.01	0.01	9.8 *
*Mesorhizobium*	0.1	0.6	0.01	8.2 *
*Chitinophaga*	0.01	0.01	0.01	3.1 *

* The main five genera in every sample.
